# Associations between paediatric fatigue and eating behaviours

**DOI:** 10.1002/osp4.422

**Published:** 2020-06-17

**Authors:** Megan M. Oberle, Elise F. Northrop, Carolyn T. Bramante, Kyle D. Rudser, Amy C. Gross, Aaron S. Kelly

**Affiliations:** ^1^ Department of Pediatrics University of Minnesota Medical School Minneapolis Minnesota USA; ^2^ Center for Pediatric Obesity Medicine University of Minnesota Medical School Minneapolis Minnesota USA; ^3^ Department of Medicine University of Minnesota Medical School Minneapolis Minnesota USA; ^4^ Division of Biostatistics, School of Public Health University of Minnesota Minneapolis Minnesota USA

**Keywords:** Eating behaviours, fatigue, paediatrics, quality of life

## Abstract

**Background:**

In adults, poor sleep quality is associated with increased obesogenic eating behaviours; less is known about this relationship in youth. The objectives of this study were to assess the strength of association between fatigue‐related quality of life (QoL) and eating behaviours among youth and to describe the associations in participants with percent body fat (%BF) above and below the 90th percentile for sex and age.

**Methods:**

Caregiver‐reported measures of fatigue (Pediatric QoL Multidimensional Fatigue Scale) and eating behaviours (Child Eating Behaviour Questionnaire) were obtained from participants aged 8–17 years. %BF was measured by iDXA and grouped by sex‐ and age‐specific percentiles. Multiple linear regression adjusting for age, sex and race/ethnicity was used.

**Results:**

Of the 352 participants (49% male), 44.6% had %BF >90th percentile. General, sleep/rest and cognitive fatigue QoL was inversely associated with food approach behaviours: food responsiveness, enjoyment of food, emotional overeating and desire to drink. For participants with %BF >90th percentile, higher general fatigue QoL was associated with higher satiety responsiveness (0.13; 95% confidence interval [CI 0.03, 0.24]). For participants with %BF ≤90th percentile, higher general fatigue QoL was associated with less satiety responsiveness (−0.16; 95% CI [−0.31, −0.01]).

**Conclusion:**

Less fatigue symptoms were associated with less behaviours associated with food approach among paediatric participants. For participants with %BF >90th percentile, less symptoms of general fatigues corresponded with more satiety. Though causation has yet to be established, youth with elevated %BF should be screened for fatigue symptoms and offered counselling on sleep hygiene or a sleep medicine referral to help mitigate weight gain.

## 1 INTRODUCTION

Approximately 20% of adolescents aged 6–19 years in the United States have obesity, defined as body mass index (BMI) >95th percentile for age and sex. Obesity significantly increased the risk of type 2 diabetes, cardiovascular disease, cancer and all‐cause mortality in adulthood.^1^, ^2^, ^3^, ^4^, ^5^, ^6^, ^7^, ^8^, ^9^, ^10^, ^11^, ^12^, ^13^, ^14^, ^15^, ^16^, ^17^, ^18^ Obesity also increased the risk of obstructive sleep apnoea (OSA)—youth with obesity are six times more likely to have OSA than youth with normal weight.^19^ In both adults and youth, poor sleep quality and shortened sleep duration have been associated with a higher risk of obesity. ^20^, ^21^, ^22^, ^23^, ^24^, ^25^, ^26^, ^27^, ^28^, ^29^, ^30^ OSA may be a reason for poor sleep quality in youth with obesity, but poor sleep quality may also be a contributing factor in excess weight gain.

Possible factors contributing to the association between poor sleep quality and obesity include the effect of sleep quality on motivation/cognitive fatigue, obesogenic eating behaviours and caloric intake in children.^31^, ^32^ Both cognitive fatigue (subjective feeling of exhaustion that follows sustained cognitive demands) and decreased motivation/anhedonia were associated with elevated BMI, independent of sex and age, in youth with obesity.^33^ Reduced motivation and cognitive fatigue may decrease the likelihood of engaging in certain behaviours such as routine physical activity and choosing healthy foods. For example, adolescents with disrupted sleep/wake schedules with late bedtime/late rise patterns of sleep were more likely to engage in additional screen time and less physical activity than youth with early bedtime/early rise patterns.^34^ Additionally, poor sleep quality and decreased sleep duration have been associated with obesogenic eating behaviours, such as emotional eating and food responsiveness in both youth and adults.^31^, ^35^, ^36^ Sleep restriction has also been associated with increased snacking, increased consumption of carbohydrates, fat, salt and sugar, and larger portions in both youth and adults.^37^, ^38^, ^39^, ^40^, ^41^, ^42^, ^43^, ^44^ Fatigue symptoms are not only associated with sleep quality or duration (sleep‐/rest‐related fatigue), but also can be classified as cognitive fatigue or general fatigue (i.e., feeling physically weak, feeling too tired to do things, trouble starting things).^45^ Clarifying the association between fatigue‐related quality of life (QoL) and specific eating behaviours may help elucidate the fatigue–obesity relationship as well as inform targeted behavioural modification strategies in youth with overweight, obesity and/or a rapidly increasing body fat percentage (%BF).

The objectives of this study were to (1) assess the strength of association between three subscales of fatigue‐related QoL (general, sleep/rest and cognitive fatigue) and eating behaviours among youth and (2) describe the associations between fatigue QoL and eating behaviours in children with %BF above and below the age‐ and sex‐specific 90th percentile. The a priori hypothesis was that general, sleep/rest and cognitive fatigue QoL subscales would be inversely associated with food approach behaviours (food responsiveness, emotional overeating. enjoyment of food and desire to drink). That is, higher fatigue QoL score (less fatigue symptoms) would be associated with less endorsement of food approach behaviours. Participants with %BF >90th percentile were hypothesized to have more food approach behaviours significantly associated with lower general, sleep/rest and cognitive fatigue QoL than participants with % BF ≤ 90th percentile.

## 2 MATERIALS AND METHODS

### 2.1 Study design and participants

This report was a secondary analysis of data collected as part of a cross‐sectional study of vascular health among children and adolescents with a range of BMI and %BF values.^46^ Children and adolescents (age range: 8–17 years old) were enrolled in the study. Exclusion criteria for the parent study included the following: (1) obesity from a known genetic cause (e.g., Prader–Willi); (2) history of bariatric surgery; (3) current or recent (within 3 months) use of medications known to affect endothelial health such as statins, angiotensin‐converting enzyme (ACE)‐inhibitors, peroxisome proliferator‐activated receptor (PPAR)‐gamma agonists and third‐generation beta blockers; (4) illness or significant injury in previous 2 weeks; (5) type 1 diabetes mellitus; (6) familial hypercholesterolaemia; (7) chronic kidney disease/end‐stage renal disease; (8) Kawasaki disease; (9) autoimmune inflammatory diseases; and (9) congenital heart disease. Participants with complete QoL, eating behaviour and %BF data were included in this analysis. All participants and parents/guardians provided verbal and written informed assent and consent, respectively. This parent study was approved by the University of Minnesota Institutional Review Board.

### 2.2 Measures

#### 2.2.1 Demographics

Participant age, sex and race/ethnicity were reported by the parent/guardian of the paediatric participant at baseline.

#### 2.2.2 Anthropometrics and %BF

Height and weight measurements were obtained using a wall‐mounted stadiometer and an electronic scale, respectively. The mean of three separate measurements of height and weight was used. BMI was calculated as the body weight in kilogrammes divided by the height in meters squared. %BF and fat‐free mass were determined by dual energy x‐ray absorptiometry (iDXA GE Healthcare), using standard positioning techniques, and were conducted and analysed by trained staff.^47^ Participants were separated into two groups based on %BF measured by iDXA: (1) participants ≤90th percentile for %BF and (2) participants >90th percentile for %BF. %BF percentile cut‐points were determined using previously published data on nationally representative %BF percentiles for youth aged 8–20 years old.^48^ In this previous analysis, 81% of male and 86% of female youth with Class 1 obesity (BMI >95th to <120th of the 95th percentile) were above the 90th percentile for %BF.^48^ There was a decrease in the proportion of youth with Class I obesity as the %BF threshold decreased from the 85th percentile (53% male, 60% female) and 75th percentile (33% male, 37% female).^48^ Therefore, the 90th percentile for %BF was used to in our study to describe a larger proportion of participants with both excess adiposity and Class I obesity.

#### 2.2.3 Pediatric Quality of Life Fatigue Scale

The Pediatric Quality of Life Multidimensional Fatigue Scale (PedsQL MDFS™) questionnaire was completed by the parent/guardian of the paediatric participant and encompassed three subscales: general, sleep/rest and cognitive fatigue symptoms.^49^ The PedsQL MDFS™ is an 18‐item questionnaire, which uses a 5‐point Likert scale from *never* (0) to *almost always* (4); items were reversed scored and linearly transformed to a 0–100 scale.^49^ Higher PedsQL MDFS™ score indicated higher QoL (or less fatigue symptoms) reported by the parent/guardian.^49^ Questions pertaining to general fatigue included asking the parent/guardian how often the paediatric participant felt physically weak, felt too tired to do things or had trouble starting things. Questions pertaining to sleep/rest fatigue included how often the paediatric participant slept a lot, had difficulty sleeping through the night, took a lot of naps or felt tired when waking up in the morning. Questions pertaining to cognitive fatigue included how often the paediatric participant found it hard to pay attention, remember what someone told them or remember more than one thing at a time. The PedsQL MDFS™ has been widely used in multiple paediatric disease states, including obesity, and been well validated in the paediatric population.^23^, ^42^, ^45^

#### 2.2.4 Child Eating Behaviour Questionnaire

Parent/guardians of the participants completed the Child Eating Behaviour Questionnaire (CEBQ), a validated 35‐item questionnaire designed to assess a child's eating style.^50^ The CEBQ was a caregiver‐reported measure with each item rated on a 5‐point Likert scale that ranges from *never* (1) to *always* (5). The CEBQ comprised eight domains that are considered either food approach behaviours (food responsiveness, emotional overeating, enjoyment of food and desire to drink) or food avoid behaviours (satiety responsiveness, slowness in eating, emotional undereating and food fussiness). A higher CEBQ domain score indicated greater endorsement of the specific eating behaviour by the caregiver.^51^

### 2.3 Statistical analysis

Data from all participants with PedsQL MDFS™, CEBQ and non‐missing %BF measurements were analysed (*n* = 352). Descriptive statistics using mean and standard deviation or *N* and percent summarized the cohort. Multiple linear regression using robust standard errors was performed to evaluate associations between PedsQL MDFS™ scale score and CEBQ eating domain score for the entire cohort adjusting for age, sex, race and ethnicity. Multiple linear regression using robust standard errors was performed to evaluate associations between PedsQL MDFS™ scale score and CEBQ eating domain score in both %BF groups by use of an interaction term and adjusting for age, sex, race and ethnicity. %BF groups were defined as those above tor those at or below the age‐ and sex‐specific 90th %BF percentile.^46^ Because the analysis by %BF cut‐point was a secondary objective and because we sought to limit the use of multiple comparisons, we intentionally did not directly compare associations between %BF groups. The PedsQL MDFS™ scale scores were scaled by a factor of 25 for more easily interpretable results. All statistical calculations were performed on R 3.5.0. Statistical significance was defined as *p* value <0.05.

## 3 RESULTS

### 3.1 Participant characteristics

Three hundred fifty‐two participants (48.9% male, 78.0% White, 11.4% Latino/Hispanic) were included in this analysis (Table 1). The mean age was 12.8 ± 2.7 years. Thirty‐nine percent of participants had normal weight (BMI <85th percentile), 6% overweight (BMI ≥85th to <95th percentile), 21% Class I obesity (BMI ≥95th percentile to <120% of the 95th percentile), 20% Class II obesity (BMI ≥120% of the 95th percentile to <140% of the 95th percentile) and 14% Class III obesity (BMI ≥140% of the 95th percentile). Table 1 describes the demographic characteristics of all participants in the study and by group above and below the age‐ and sex‐specific 90th percentile %BF.

**TABLE 1:** Participant demographics and caregiver‐reported eating behaviours and fatigue quality of life by %BF category with *N* (%) or mean (*SD*)

Covariate	Overall	≤90th percentile %BF	>90th percentile %BF
	*N* = 350	*N* = 184	*N* = 166
Male	171 (48.9%)	101 (54.9%)	70 (42.2%)
Race			
Black	34 (9.7%)	18 (9.8%)	16 (9.6%)
White	273 (78.0%)	149 (81.0%)	124 (74.7%)
Other	43 (12.3%)	17 (9.2%)	26 (15.7%)
Latino/Hispanic	40 (11.4%)	13 (7.1%)	27 (16.3%)
Age (years)	12.8 (2.7)	12.5 (2.48)	13.1 (2.91)
BMI (kg/m^2^)	26.5 (8.73)	20.2 (4.21)	33.5 (6.89)
Percentage of 95th percentile for BMI	104 (30.5)	80.8 (14.7)	131 (20.2)
%BF	36.9 (11.4)	27.7 (7.17)	47.1 (4.71)
Caregiver‐reported Child Eating Behaviour Questionnaire score			
Food approach behaviours:			
Food responsiveness	2.91 (0.96)	2.46 (0.8)	3.41 (0.88)
Emotional overeating	2.56 (0.99)	2.12 (0.81)	3.04 (0.95)
Enjoyment of food	3.97 (0.7)	3.79 (0.69)	4.17 (0.66)
Desire to drink	2.52 (0.98)	2.29 (0.93)	2.78 (0.97)
Food avoid behaviours:			
Satiety responsiveness	2.45 (0.66)	2.68 (0.65)	2.20 (0.56)
Slowness in eating	2.33 (0.73)	2.48 (0.75)	2.16 (0.68)
Emotional undereating	2.59 (0.7)	2.62 (0.77)	2.56 (0.62)
Food fussiness	2.70 (0.86)	2.70 (0.83)	2.69 (0.91)
Caregiver‐reported Pediatric Quality of Life Multidimensional Fatigue Scale score			
General fatigue	73.6 (19.2)	78.2 (16.3)	64.9 (22.1)
Sleep/rest fatigue	71.9 (20.4)	79.6 (15.6)	66.9 (20.6)
Cognitive fatigue	72.0 (22.2)	75.7 (20.9)	67.9 (23.0)

Abbreviations: %BF, percent body fat; BMI, body mass index.

### 3.2 Associations between general QoL and eating behaviours

#### 3.2.1 General fatigue subscale

For the entire cohort, general fatigue QoL was inversely associated with all four food approach behaviours: food responsiveness (−0.45; 95% confidence interval [CI −0.57, −0.33]), emotional overeating (−0.48; 95% CI [−0.61, −0.37]), enjoyment of food (−0.12; 95% CI [−0.21, −0.02]) and desire to drink (−0.34; 95% CI [−0.47, −0.22]; Table 2). That is, higher QoL (less general fatigue symptoms) corresponded with lower endorsement of food responsiveness, emotional overeating, enjoyment of food and desire to drink behaviours. General fatigue QoL was positively associated with the food avoid behaviour, satiety responsiveness (0.11; 95% CI [0.02, 0.20]); higher QoL (lower general fatigue symptoms) corresponded with higher satiety responsive scores (ceasing eating when full). General fatigue QoL was positively associated with two other food avoid behaviours: emotional undereating (−0.10; 95% CI [−0.19, −0.01]) and food fussiness (−0.12; 95% CI [−0.23, 0.00]).

**TABLE 2:** Associations between fatigue QoL and eating behaviours

Eating behaviours (outcome)	Fatigue QoL (covariate)	Increase per 25 point increase in fatigue QoL (95% CI)
Food approach behaviours		
Food responsiveness	General fatigue	−0.45 (−0.57, −0.33)^a^
	Sleep/rest fatigue	−0.44 (−0.58, −0.30)^a^
	Cognitive fatigue	−0.29 (−0.41, −0.18)^a^
Emotional overeating	General fatigue	−0.49 (−0.61, −0.37)^a^
	Sleep/rest fatigue	−0.57 (−0.71, −0.44)^a^
	Cognitive fatigue	−0.30 (−0.41, −0.19)^a^
Enjoyment of food	General fatigue	−0.12 (−0.21, −0.02)^a^
	Sleep/rest fatigue	−0.12 (−0.23, −0.01)^a^
	Cognitive fatigue	−0.11 (−0.19, −0.02)^a^
Desire to drink	General fatigue	−0.34 (−0.47, −0.22)^a^
	Sleep/rest fatigue	−0.31 (−0.45, −0.16)^a^
	Cognitive fatigue	−0.21 (−0.32, −0.09)^a^
Food avoid behaviours		
Satiety responsiveness	General fatigue	0.11 (0.02, 0.20)^a^
	Sleep/rest fatigue	0.09 (−0.01, 0.19)
	Cognitive fatigue	0.01 (−0.07, 0.09)
Slowness in eating	General fatigue	0.09 (−0.01, 0.19)
	Sleep/rest fatigue	0.06 (−0.05, 0.18)
	Cognitive fatigue	−0.03 (−0.12, 0.06)
Emotional undereating	General fatigue	−0.10 (−0.19, −0.01)^a^
	Sleep/rest fatigue	−0.10 (−0.21, 0.01)
	Cognitive fatigue	−0.02 (−0.11, 0.06)
Food fussiness	General fatigue	−0.12 (−0.23, 0.00)^a^
	Sleep/rest fatigue	−0.10 (−0.23, 0.04)
	Cognitive fatigue	0.00 (−0.10, 0.11)

*Note.* Multiple linear regression models adjusted for sex, age, race and ethnicity. Confidence intervals were calculated using robust standard errors.

Abbreviations: CI, confidence interval; QoL, quality of life.

a Significant *p* values (<0.05).

For participants with %BF ≤90th percentile, general fatigue QoL score was inversely associated with three food approach behaviours: food responsiveness (−0.33; 95% CI [−0.51, −0.15]), emotional overeating (−0.48; 95% CI [−0.66, −0.29]) and desire to drink (−0.40; 95% CI [−0.60,‐0.19]; Table 3). General fatigue QoL score was also inversely associated with three food avoid behaviours: satiety responsiveness (−0.16; 95% CI [−0.31, −0.03]), emotional undereating (−0.24; 95% CI [−0.39, −0.008]) and food fussiness (−0.25; 95% CI [−0.44, −0.05]), meaning higher QOL score (less symptoms) corresponded with less endorsement of satiety responsiveness, emotional undereating and food fussiness for participants with %BF ≤90th percentile.

**TABLE 3:** Associations between fatigue QoL and eating behaviours by %BF category

Eating behaviours (outcome)	Fatigue QoL (covariate)	≤90th percentile %BF	>90th percentile %BF
Food approach behaviours			
Food responsiveness	General fatigue	−0.33 (−0.51, −0.15)^a^	−0.28 (−0.42, −0.14)^a^
	Sleep/rest fatigue	−0.33 (−0.53, −0.14)^a^	−0.22 (−0.38, −0.06)^a^
	Cognitive fatigue	−0.19 (−0.33, −0.05)^a^	−0.20 (−0.34, −0.07)^a^
Emotional overeating	General fatigue	−0.48 (−0.66, −0.29)^a^	−0.29 (−0.44, −0.15)^a^
	Sleep/rest fatigue	−0.51 (−0.71, −0.31)^a^	−0.39 (−0.55, −0.23)^a^
	Cognitive fatigue	−0.30 (−0.45, −0.16)^a^	−0.14 (−0.28, 0.01)
Enjoyment of food	General fatigue	0.10 (−0.05, 0.25)	−0.14 (−0.26, −0.02)^a^
	Sleep/rest fatigue	0.07 (−0.09, 0.24)	−0.11 (−0.24, 0.02)
	Cognitive fatigue	0.05 (−0.07, 0.17)	−0.17 (−0.29, −0.06)^a^
Desire to drink	General fatigue	−0.40 (−0.60, −0.19)^a^	−0.21 (−0.37, −0.05)^a^
	Sleep/rest fatigue	−0.45 (−0.68, −0.23)^a^	−0.09 (−0.27, 0.09)
	Cognitive fatigue	−0.21 (−0.37, −0.05)^a^	−0.11 (−0.27, 0.04)
Food avoid behaviours			
Satiety responsiveness	General fatigue	−0.16 (−0.31, −0.03)^a^	0.13 (0.03, 0.24)^a^
	Sleep/rest fatigue	−0.09 (−0.24, 0.06)	0.03 (−0.09, 0.16)
	Cognitive fatigue	−0.13 (−0.24, −0.03)^a^	0.03 (−0.07, 0.14)
Slowness in eating	General fatigue	−0.15 (−0.31, 0.01)	0.15 (0.02, 0.27)^a^
	Sleep/rest fatigue	−0.06 (−0.23, 0.12)	0.03 (−0.11, 0.17)
	Cognitive fatigue	−0.17 (−0.29, −0.05)^a^	0.04 (−0.08, 0.16)
Emotional undereating	General fatigue	−0.24 (−0.39, −0.08)^a^	−0.05 (−0.18, 0.07)
	Sleep/rest fatigue	−0.15 (−0.32, 0.02)	−0.11 (−0.25, 0.03)
	Cognitive fatigue	−0.12 (−0.24, 0.00)	0.05 (−0.06, 0.17)
Food fussiness	General fatigue	−0.25 (−0.44, −0.05)^a^	−0.06 (−0.21, 0.09)	
	Sleep/rest fatigue	−0.21 (−0.42, 0)	−0.05 (−0.22, 0.12)	
	Cognitive fatigue	−0.07 (−0.22, 0.08)	0.08 (−0.07, 0.22)	

*Note.* Multiple linear regression models adjusted for sex, age, race and ethnicity. Confidence intervals were calculated using robust standard errors.

Abbreviations: %BF, percent body fat; QoL, quality of life.

a Significant *p* values (<0.05).

For participants with %BF >90th percentile, general fatigue QoL score was inversely associated with all four food approach behaviours: food responsiveness (−0.28; 95% CI [−0.42, −0.14]), emotional overeating (−0.29; 95% CI [−0.44, −0.15]), enjoyment of food (−0.14; 95% CI [−0.26, −0.02]) and desire to drink (−0.21; 95% CI [−0.37, −0.05]). General fatigue QoL was positively associated with two food avoid behaviours: satiety responsiveness and slowness in eating for participants with %BF >90th percentile. That is, higher general fatigue QoL score (less symptoms) corresponded with higher satiety responsiveness (0.13; 95% CI [0.03, 0.24]) and slowness in eating (0.15; 95% CI [0.02, 0.27]).

#### 3.2.2 Sleep/rest fatigue scale

For the entire cohort, sleep/rest fatigue QoL score was inversely associated with all four food approach behaviours: food responsiveness (−0.44; 95% CI [−0.57, −0.30]), emotional overeating (−0.57; 95% CI [−0.71, −0.44]), enjoyment of food (−0.12; 95% CI [−0.23, −0.01]) and desire to drink (−0.31; 95% CI [−0.45, −0.16]), meaning higher sleep/rest fatigue QOL (less symptoms) corresponds with less endorsement of food approach behaviours.

For participants with %BF ≤90th percentile, sleep/rest fatigue QoL score was inversely associated with three food approach behaviours: food responsiveness (−0.33; 95% CI [−0.51, −0.15]), emotional overeating (−0.51; 95% CI [−0.71, −0.31]) and desire to drink (−0.45; 95% CI [−0.68, −0.23]). There were no significant associations between sleep/rest fatigue QOL score and food avoid behaviours for participants with %BF ≤90th percentile.

For participants with %BF >90th percentile, sleep/rest fatigue QoL score was inversely associated with two food approach behaviours: food responsiveness (−0.22; 95% CI [−0.38, −0.06]) and emotional overeating (−0.39; 95% CI [−0.55, −0.23]). There were no significant associations between sleep/rest fatigue QOL score and food avoid behaviours for participants with %BF >90th percentile.

#### 3.2.3 Cognitive fatigue scale

For the entire cohort, cognitive fatigue QoL was inversely associated with all four food approach behaviours: food responsiveness (−0.29; 95% CI [−0.41, −0.18]), emotional overeating (−0.30; 95% CI [−0.41, −0.19]), enjoyment of food (−0.11; 95% CI [−0.19, −0.02]) and desire to drink (−0.21; 95% CI [−0.32, −0.09]). That is, higher cognitive fatigue QOL (less symptoms) corresponds with less endorsement of food approach behaviours.

For participants with %BF <90th percentile, cognitive fatigue QoL score was inversely associated with three food approach behaviours: food responsiveness (−0.19; 95% CI [−0.33, −0.05]), emotional overeating (−0.30; 95% CI [−0.45, −0.16]) and desire to drink (−0.21; 95% CI [−0.37, −0.05]). Cognitive fatigue QoL score was also inversely associated with two food avoid behaviours: satiety responsiveness (−0.13; 95% CI [−0.24, −0.03]) and slowness in eating (−0.17; 95% CI [−0.29, −0.05]). That is, higher cognitive fatigue QOL score (less symptoms) corresponded with less endorsement of food avoid behaviours in participants with %BF <90th percentile.

For participants with %BF >90th percentile, cognitive fatigue QoL score was inversely associated with two food approach behaviours: food responsiveness (−0.20; 95% CI [−0.34, −0.07]) and enjoyment of food (−0.17; 95% CI [−0.29, −0.06]). There were no significant associations between cognitive fatigue QOL score and food avoid behaviours for participants with %BF >90th percentile.

## 4 DISCUSSION

This cross‐sectional study evaluated the association between three subscales of fatigue‐related QoL (general, sleep/rest and cognitive fatigue) and eating behaviours and characterized the association in among youth with %BF above and below the age‐ and sex‐specific 90th percentile. Less general, sleep‐/rest‐related and cognitive fatigue were associated with lower endorsement of all four food approach behaviours (food responsiveness, emotional overeating, enjoyment of food and desire to drink) in the overall cohort. Additionally, associations between general fatigue and food avoid behaviours (satiety responsiveness, slowness in eating, emotional undereating and food fussiness) differed in directionality for participants with %BF above and below the age‐ and sex‐specific 90th percentile.

Improvement in general, sleep‐/rest‐related and cognitive fatigue may be a lifestyle management focus for children with rapidly increasing BMI (even with a %BF <90th percentile) as addressing food approach behaviours may taper rapid weight gain. For example, specific food approach behaviours, such as desire to drink and emotional overeating, may serve as targets for higher yield behaviour interventions for patients with endorsement of general, sleep‐/rest‐related or cognitive fatigue symptoms regardless of BMI or %BF. Endorsement of desire to drink behaviour has been associated with a higher preferences for soda, fruit juice and milk and higher consumption of these beverages in children.^52^ Excess fruit juice and sugar‐sweetened beverage (SSB) consumption is an important contributor in the development of obesity among children and adolescents, and limiting access to these beverages is an important dietary intervention for weight management.^53^, ^54^, ^55^ Additionally, health care providers can provide targeted behavioural strategies in patients who endorse emotional overeating to decrease emotion‐driven eating patterns, such as mindful eating techniques and stress reduction.^56^ Focusing on decreasing SSB consumption or stress reduction are concrete lifestyle management approaches that can be offered in a clinical setting in addition to evaluating and treating causes of general, cognitive and sleep‐/rest‐related fatigue (i.e., depression, poor sleep hygiene or sleep apnoea).

An important difference in the association between fatigue subscales and food avoid behaviours (satiety responsiveness, slowness in eating, emotional undereating and food fussiness) for patients with %BF above and below the age‐ and sex‐specific 90th percentile was also described in this study. For participants with %BF >90th percentile, less general fatigue symptoms corresponded with more endorsement of satiety responsiveness and slowness in eating. Investigating the aetiology of and improving fatigue in patients with an elevated %BF may be an important intervention target, which may result in slowed eating speed and improved satiety response, leading to slowed weight gain or weight loss. Conversely, for participants with %BF ≤90th percentile, less general fatigue symptoms corresponded with less satiety responsiveness, emotional undereating and food fussiness. Taken together, these findings regarding food avoid behaviours suggest that youth with a lower %BF avoided food more when more fatigued, whereas youth with a higher %BF avoided food more when less fatigued. Evaluating for causes of general fatigue and treating these underlying causes may promote satiety responsiveness and slowness in eating for participants with %BF >90th percentile. It is important to note that fatigue and eating behaviours are multifactorial, and other interventions, such as parenting techniques, sleep environment, and biological mechanisms, should be evaluated to address weight management. Additionally, youth with lower %BF may have different genetic or other biological factors than youth with a %BF >90th percentile, which contribute to their current %BF as well as their appetite and eating behaviour response to fatigue, which were not measured in this study.

This study had some limitations. The caregiver, not the paediatric participants, reported both the child eating behaviours and quality of life scales. Given that the average age of the participant was 12 years, caregivers may not be aware of an adolescent's feelings of fatigue, sleep quality or eating behaviours outside of the home. The CEBQ has been developed for, and historically used in, a younger age range. However, it is an accepted measure of child eating behaviours and has been used in adolescent youth.^57^, ^58^, ^59^ Additionally, participants' ages in this study ranged from age 8–17 years, and sleep and eating behaviours, as well as parental involvement affecting behaviours, can vary widely in this age range. However, regression models were adjusted for age. Our results demonstrating an association between food approach behaviours and fatigue QoL subscale added to the previous literature that suggests sleep quality and duration may lead to increased appetite stimulation and caloric intake despite this limitation. ^37^, ^38^, ^39^, ^43^, ^44^, ^60^, ^61^ The CEBQ and PedsQL MDFS™ were conducted among an English‐speaking only population, which makes it difficult to account for some of the social and cultural determinants of eating behaviours and sleep patterns. Importantly, as this was not a longitudinal study, we were unable to evaluate causality as to whether fatigue lends to certain eating behaviours or will result in rapid gain weight. Other factors, such as parenting techniques, environment and biological mechanisms, likely were part of this association.

The results of this study expanded upon the current literature evaluating the association between sleep quality and obesity, offering insight into which eating behaviours are affected by not only sleep‐/rest‐related fatigue, but also general and cognitive fatigue. Furthermore, this study provided evidence that less general fatigue symptoms correspond with increased endorsement of satiety responsiveness and slowness in eating behaviours in children with %BF >90th percentile for age and sex. Therefore, based on our findings, when children were well rested, they were more able to avoid unnecessary food intake, which may aid in weight management among children with elevated %BF. Therefore, screening for poor sleep quality and other fatigue‐related symptoms, counselling on sleep hygiene and evaluating for other causes of fatigue are recommended for youth with overweight, obesity or a rapidly increasing BMI percentile. Similarly, evaluation and targeted behavioural intervention for specific obesogenic eating behaviours, such as desire to drink or emotional overeating, is also recommended when fatigue is identified in youth with overweight or obesity. In conclusion, high fatigue symptoms were significantly associated with increased food approach behaviours in youth. Future efforts aimed at characterizing the potential causal pathways between fatigue and poor sleep quality leading to unhealthy eating behaviours may inform more targeted and effective prevention and treatment strategies for obesity in youth.

## CONFLICTS OF INTEREST

Dr Kelly receives research support (drug/placebo) from Astra Zeneca Pharmaceuticals and serves as a consultant for Novo Nordisk, WW and Vivus Pharmaceuticals but does not accept personal or professional income for these activities. Dr Oberle receives research support from Vivus Pharmaceuticals. No other competing financial interests exist.

## FUNDING

Funding for this project was provided by the National Institutes of Health (NIH; Bethesda, MD), the National Heart, Lung, and Blood Institute/NIH (R01HL110957, awarded to A.S.K.), the National Center for Advancing Translational Sciences/NIH (UL1TR000114), and National Institute of Diabetes and Digestive and Kidney Diseases (NIDDK)/NIH NORC (grant P30 DK050456).

## AUTHOR CONTRIBUTIONS

MMO, EFN, KDR and ASK collaborated in formulating the research question, analysing the data and writing the manuscript. EFN and KDR also performed the statistical analysis. CTB and ACG collaborated in the manuscript writing. All authors were involved in writing the paper and had final approval of the submitted and published versions.

## INFORMED CONSENT AND ETHICAL CONSIDERATIONS

This study was conducted according to the guidelines laid down in the Declaration of Helsinki, and the University of Minnesota Institutional Review Board approved all procedures involving human subjects.
